# Targeted Transition Readiness Workshops for Pediatric Brain Tumor Survivors: Feasibility, Acceptability, and Preliminary Effects

**DOI:** 10.3390/curroncol32010034

**Published:** 2025-01-08

**Authors:** Julie Carrier, Tziona Lugasi, Nathalie Labonté, Carole Provost, Andrea Saragosti, Claire Longpré, Bénédicte Koukoui, Émilie Régnier-Trudeau, Serge Sultan, Hallie Coltin, Sébastien Perreault, Marco Bonanno, Leandra Desjardins

**Affiliations:** 1 Department of Psychology, Université de Montréal, Montréal, QC H3T 1J4, Canada; 2 Pediatric Oncology, Sainte-Justine University Health Center, Montréal, QC H3T 1C5, Canada; 3 Department of Pediatrics, Université de Montréal, Montréal, QC H3T 1J4, Canada

**Keywords:** transition readiness, pediatric brain tumor, survivorship, pilot studies

## Abstract

Pediatric brain tumor survivors (PBTS) are at risk for late effects related to their diagnosis and treatment. Long-term medical follow-ups are deemed essential, implying a transition from pediatric to adult healthcare settings. This pilot study aims to assess the feasibility, acceptability, and preliminary effects of a targeted transition readiness intervention for PBTS. The program consisted of three hybrid workshops that targeted disease-related self-management skills, social skills, and cognitive functioning, as well as parallel workshops for their caregivers. The feasibility and acceptability were assessed through recruitment, retention, and satisfaction rates. Preliminary effects were primarily assessed via a pre/post assessment of transition readiness skills using the Transition Readiness Assessment (TRAQ) questionnaire. Among the eligible dyads, 12 (38%) consented to participate. Ten dyads participated in at least two workshops, and six dyads participated in all workshops. Overall, the participants were satisfied with the intervention (parents = 86%; PBTS = 73%). Although not statistically significant, a clinically relevant post-workshop increase in transition readiness skills was observed for PBTS (d = 0.36) and their caregivers (d = 0.25). The results suggest the relevance of the intervention and encourage further developments. Adjustments are needed to optimize reach and efficacy. The workshops have the potential to be adapted to be more accessible and shorter.

## 1. Introduction

The population of pediatric brain tumor survivors (PBTS) is growing due to rising incidence rates as well as improved treatment approaches [1,2]. However, survival can be accompanied by physical, psychosocial, and cognitive sequelae related to their disease and treatment, which can increase over time and can be late in onset [2,3,4,5,6,7]. It is estimated that more than two-thirds of PBTS have at least one physical sequelae, such as headaches, dizziness, loss of vision and hearing, sleeping problems, or secondary neoplasms [5,6,8]. Regarding psychosocial sequelae, individuals in this population frequently encounter difficulties with social skills, which can lead to difficulties in relationships [9,10,11,12,13]. For instance, difficulties with social communication include challenges in social information processing as well as asking for information [14,15]. Regarding neurocognitive sequelae, PBTS can present various impairments in cognitive function [5,12,16,17]. These may include difficulties with attention, information processing speed, memory, planning, and organization [5,7,12,16].

Due to the various potential late effects, PBTS require long-term medical follow-up to maintain a good level of health and quality of life [4,5,18,19]. Proper preparation for transfer to the adult healthcare setting is associated with several long-term benefits, including improved adherence to care, enhanced self-care skills, and a better experience with and use of healthcare services [20,21]. Ultimately, better adherence to long-term care in adult settings can contribute to the identification and early intervention of potential sequelae, leading to a better quality of life, or even survival, of PBTS [18,21]. It is, therefore, essential to ensure an optimal preparation for transition between pediatric and adult medical care settings [22,23,24].

Transition readiness is the gradual process in which the adolescent or young adult develops skills and knowledge that allow for successful transfer between pediatric care and adult healthcare contexts [25]. The goal of transition readiness is to optimize patients’ overall and long-term functioning by enabling them to take on the responsibilities typical of adulthood, such as attending medical appointments independently [22,24]. Thus, the process of preparing for transition is centered on the development of autonomy [26,27]. Adult health care differs from pediatric health care notably by attributing patients a greater responsibility and independence, implying the ability to access services, express their needs, and have good medical decision-making skills [22,24,28]. The current recommendation is to begin the transition process from the age of 14 in adolescents with chronic illnesses [22,29]. Unfortunately, more than 80% of adolescents and young adults report not receiving the necessary planning to facilitate this process [30].

The various sequelae related to brain tumors and their treatment, particularly cognitive and social ones, can hinder the acquisition of necessary transition readiness skills [31]. PBTS have been noted to experience more challenges than other populations with pediatric chronic disease in terms of disease self-management skills, which is an important component of transition readiness [6,7,26,27,32]. Also, PBTS’ potential challenges in social skills, such as the ability to ask questions [33], could hinder their active participation in individual medical appointments in an adult setting. In addition, neurocognitive sequelae may impact their ability to remember appointments and recall information provided [5,7,12,16]. Parental overprotection, which is a common phenomenon among PBTS families, is another potential obstacle to developing autonomy [26,34,35]. The abrupt change from family-focused care in the pediatric setting to patient-focused care in adult hospital settings (often excluding parents) can be especially difficult for young patients who have not had an opportunity to practice a more active role in the pediatric setting [24,26]. Overprotective parenting can, therefore, hinder the development of autonomy skills necessary to successfully navigate adult healthcare settings [36,37]. Furthermore, the lack of autonomy support can also be found in healthcare professionals working with pediatric populations, who may tend to overprotect young patients [38]. Autonomy-supporting behaviors of pediatric healthcare providers towards young patients are also associated with better transition readiness [39].

Several studies have focused on the development and evaluation of transition readiness interventions [18,20,21,23,40,41]. Existing transition interventions have primarily focused on self-management skills such as disease education and learning one’s health history [21,40,41]. Interventions are also primarily applied to populations of adolescents and young adults with a variety of chronic pediatric conditions [21,23,41]. Although disease self-management is an important component of transition readiness, this uniform approach does not address specific challenges faced by PBTS, such as social and neurocognitive challenges [22,24,26,27,32].

While there are interventions for the PBTS population to help them improve their neurocognitive challenges or social skills, to our knowledge, no psychosocial interventions currently exist that specifically address the transition readiness needs of PBTS [16,21,23,40,41,42,43,44,45]. The only intervention that was found in the literature regarding the transition readiness of PBTS is a mixed transition consultation with a neurosurgeon from both pediatric and adult healthcare settings, which focuses largely on the aspect of transfer in a neurosurgery department [46]. Thus, in line with the specific needs of this population and the available empirical data highlighting the association between social, neurocognitive, and disease management skills, the principal purpose of this study was to develop an intervention for PBTS and their parents that targets disease self-management, social skills, and cognitive challenges for transition readiness [6,7,12,31,33].

In accordance with current recommendations for behavioral program optimization [47], the primary objective of this study was to assess the feasibility and acceptability of the newly developed targeted transition workshops for PBTS and their parents. Secondarily, this study also aimed to explore the impact of targeted workshops on PBTS transition readiness skills and different measures related to transition readiness and the workshop content.

## 2. Materials and Methods

### 2.1. Design

A program of three workshops on transition readiness specifically designed for PBTS and their parents was created by the research team in collaboration with clinical practitioners in the hospital setting. The content and format of the workshops were co-developed and refined over nine multidisciplinary meetings with healthcare professionals and psychosocial workers who have expertise with this population (psychologist, social worker, nurse, rehabilitation professionals, and parent–partner), led by the research team at a large tertiary pediatric hospital. An intervention manual, as well as resource documents for participants, were then developed based on meeting discussions.

The specific practical skills addressed during the workshop program were selected among those detailed in the Transition Readiness Assessment Questionnaire (TRAQ) [48]. The TRAQ is the most empirically validated questionnaire for measuring transition readiness skills [49,50]. The workshop program is also based on the ABC Transition process model by Gorter [51] and the Social-ecological model of AYA (adolescents and young adults) readiness for transition (SMART) by Schwartz and colleagues [52]. Considering the impact of parental overprotection on transition readiness in PBTS, parental participation was integrated into the workshop design [36,37]. However, the workshops were held separately for PBTS and their parents to meet the specific needs of each and to support the development of PBTS’ autonomy [26,27]. The workshops were offered in a hybrid format (online and in-person) to facilitate accessibility for families living far from the hospital.

### 2.2. Participants

A sample of 12 dyads was recruited to participate in the research project. Although the workshops were offered in French or English to participants in the consent form, all dyads chose to attend the workshops in French. More details about recruitment and samples can be found in Section 3. PBTS were eligible if they had a diagnosis of a pediatric brain tumor, cancerous or not, and were in remission for at least 1 year. They also had to be 14 years of age and older, and they had to have ongoing pediatric follow-up. In terms of exclusion criteria, PBTS were required to not be in relapse or receiving palliative care. Parents were eligible if they were the parent or primary caregiver of a PBTS and met the inclusion criteria of the project. They were excluded if the PBTS did not agree to participate.

### 2.3. Procedure

The project was approved by the research ethics board of the hospital where the project took place. Recruitment was conducted over a five-month period through posters in the oncology clinic, email, and social media posts by community pediatric cancer organizations, and phone calls and in-person visits to eligible patients in the outpatient neuro-oncology and long-term follow-up clinic in the oncology center. After giving their consent, the participants completed the pre-intervention questionnaires. They were then invited to participate in the program of three workshops, with an interval of 2 months between each workshop. Participants completed a short satisfaction questionnaire after each workshop. At the conclusion of the workshop program, they again completed the pre-intervention questionnaires.

#### Workshop Schedule

Participants who attended the in-person workshops met at a neutral meeting room in the hospital, and parking costs were reimbursed. Otherwise, the format of the online workshops was synchronous by videoconference. The specific content of the workshops is detailed in Table 1. The workshops were co-facilitated by a clinician–researcher as well as a clinical professional related to the theme of the workshop. Each workshop followed the same structure. First, a period of psychoeducation related to the workshop theme was provided. This was followed by a testimonial and question and answer period with a PBTS or a parent who had transferred to the adult care setting. After a break, two practical skills related to the theme of the workshop were taught interactively. In total, the workshops lasted 1 h 30 each. At the end of each workshop, participants were invited to complete a brief workshop evaluation (satisfaction questionnaire).

### 2.4. Measures

#### 2.4.1. Socio-Demographic and Medical Data

A questionnaire was used to compile participants’ socio-demographic data (e.g., age), as well as information on PBTS’ health history (e.g., diagnosis and treatments received).

#### 2.4.2. Acceptability

Acceptability was assessed through participant satisfaction, as recommended by Bowen and collaborators [53]. A satisfaction questionnaire of 12 items with a Likert-type scale (1 = Strongly disagree, 5 = Strongly agree) and 3 open-ended questions was administered to PBTS and parents to assess satisfaction with the workshops (see Supplementary Materials). The questionnaire is an adaptation of an existing intervention satisfaction questionnaire [54]. The items relate to the format, for example, “The duration of the workshop was ideal”, and the content of the workshops, for example, “The information was useful”. The average percentages of participants who respond “Agree” and “Strongly agree” for each workshop and for the workshop program are reported for the PBTS group as well as for the parent group.

#### 2.4.3. Transition Readiness

The Transition Readiness Assessment Questionnaire (TRAQ) is a tool that measures the current level of patients’ transition readiness skills. It is intended for adolescents and young adults and can be used across a range of diagnoses [48]. The French version of the questionnaire is composed of 19 items with a Likert-type scale (1 = No, I don’t know how to do it, 5 = Yes, I always do it when necessary) [55]. The items are divided into 5 subscales, i.e., medication management, keeping appointments, tracking health problems, talking to health care providers, and managing daily activities [48,55]. The score of the global scale is obtained by calculating the average of the items [48]. The French version of the TRAQ demonstrates good reliability and validity [55].

Additional questionnaires were administered to participants to explore various aspects related to transition readiness. Both parents and PBTS completed a measure regarding PBTS self-efficacy (The General Self-Efficacy Short Scale; GSES) [56]. Parents completed a questionnaire assessing PBTS social skills (Social Skills Improvement System; SSIS), while PBTS completed the “Assertiveness” subscale only [57]. Parents also completed a questionnaire to assess PBTS executive functions (The Behavior Rating Inventory of Executive Function; BRIEF), while PBTS completed the “Plan/Organize” subscale only [58]. Finally, parents only completed a measure to assess parental overprotection (The Parent Protection Scale; PPS) [59].

### 2.5. Data Analysis

Descriptive statistics (i.e., means, standard deviations, ranges, frequencies, and sample percentages) were conducted for socio-demographic data. To assess the feasibility of targeted transition workshops with PBTS and their parents, descriptive statistics (i.e., frequencies and sample percentages) on recruitment and retention frequencies were collected. A rate greater than or equal to 50% of participants attending all three workshops and 60% attending at least 2 workshops would indicate the workshops as feasible, similar to what has already been shown for other pediatric oncology intervention programs [54,60]. To assess the acceptability of targeted transition workshops with PBTS and their parents, descriptive data were also collected on the satisfaction rate means. The research team determined that the workshop program would be considered acceptable if 75% or more of the participants indicated, on average, that they “agree” and “strongly agree” with the components of the workshop satisfaction questionnaire (Supplementary Materials) [54]. To assess the impact of targeted workshops on transition readiness, dependent samples *t*-tests were performed to examine the results of the TRAQ questionnaire. Dependent *t*-tests were also conducted on other variables that were studied in an exploratory manner (i.e., self-efficacy, social skills, executive functions, parental overprotection). The data were interpreted primarily using the effect size, i.e., Cohen’s d, which was interpreted as follows: 0.2 is considered a small effect size, 0.5 is considered a moderate effect size, and 0.8 is considered a large effect size [61]. The analyses were carried out using the SPSS software, version 29, with a significance threshold of 0.05.

## 3. Results

A sample of 12 dyads was recruited to participate in the research project. For the PBTS group, their age ranged from 15 to 19 years old (M = 17.17; SD = 1.4), time since diagnosis ranged from 4 to 14 years (M = 10.75 years; SD = 3.28), time since completion of treatment ranged from 4 to 14 years (M = 9.2; SD = 3.67), and frequency of oncology follow-up ranged from none to twice per year (M = 0.83; SD = 0.57) (See Table 2 for more details). For the parent group, participants’ ages ranged from 41 to 57 years old (M = 49.58; SD = 4.83); four (25%) were fathers, and eight (75%) were mothers.

### 3.1. Feasibility

#### 3.1.1. Recruitment

A total of 40 eligible patients were identified in the neuro-oncology clinic and the outpatient long-term clinic of our hospital. Eight families could not be reached by the tracing team, either because of missing contact information or no response to recruitment calls. Of the remaining 32 eligible families that were reached, 12 families (38%) were recruited and participated in the research project. Otherwise, 11 families (34%) indicated an interest in the project but never returned a signed consent form to the research team. Nine families (28%) actively declined to participate. The reasons for refusal cited by the families were the PBTS’ lack of interest, the family’s lack of time, and the impression that themes addressed by the workshops were not relevant to them. The research team did not receive any contact through online and poster advertising.

#### 3.1.2. Retention

Overall, 10 dyads participated in at least two workshops, representing 83% of participants, and 6 dyads participated in all workshops, representing 50% of participants. Otherwise, one dyad participated in only one workshop, and one dyad did not participate in any workshops. A total of 10 parents and 10 PBTS attended the first workshop, while 7 parents and 7 PBTS participated in the second workshop, and 7 parents and 6 PBTS were present at the third workshop.

### 3.2. Acceptability

#### Satisfaction

The results of the satisfaction survey are detailed in Table 3. In terms of overall satisfaction with the workshops, most PBTS participants (73%) rated the workshop program as “acceptable” or “very acceptable”. The workshop where PBTS expressed the most satisfaction was on social skills and peer relations (2nd workshop; 79%). The workshop where PBTS participants expressed the least satisfaction was the one pertaining to cognitive challenges and return to daily activities (3rd workshop; 61%). In terms of overall satisfaction among parents, an average of 86% of parents rated the workshop program as “acceptable” or “very acceptable”. The workshops in which parent participants expressed the most satisfaction were those on social skills and peer relations (2nd workshop; 88%) and on cognitive challenges (3rd workshop; 88%). Parent participants expressed the least satisfaction with the workshop on disease self-management (1st workshop; 83%). Notably, on average, 97% of parents and 98% of young people responded that they described the workshop program as “neutral”, “acceptable”, or “very acceptable”. These results highlight the low level of dissatisfaction (unacceptable or very unacceptable) among participants with the workshops.

**Figure 1:**
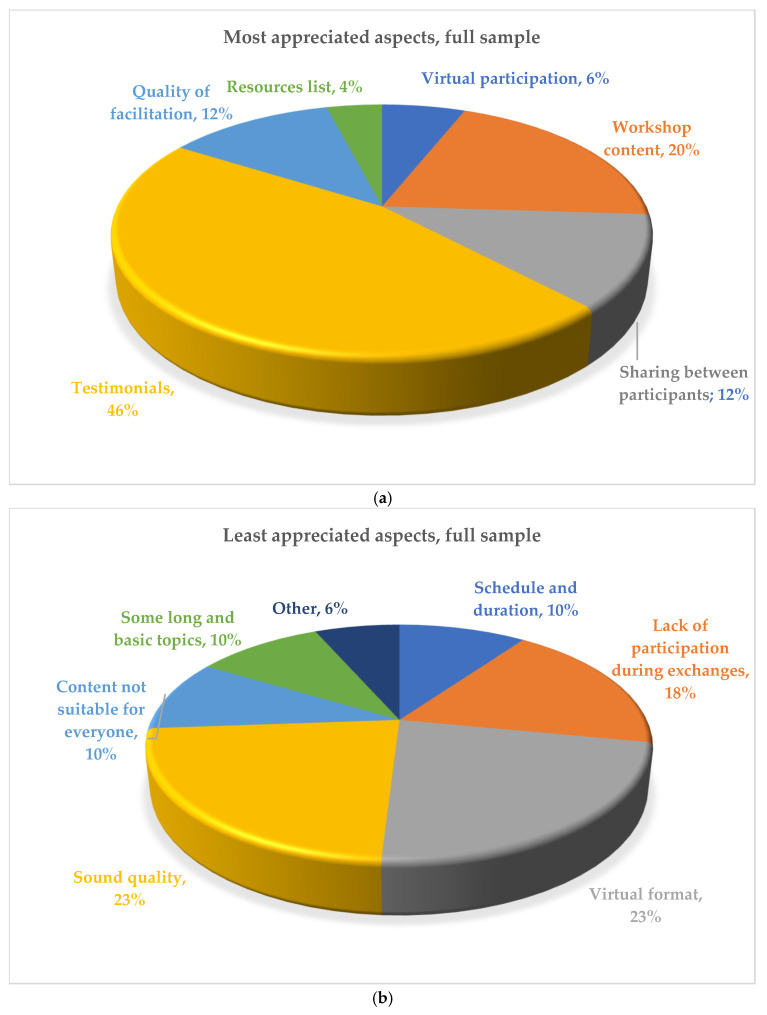
Description of the participant’s most (**a**) and least (**b**) appreciated aspects of the workshops emerged from the open-ended questions of the satisfaction questionnaire (n = 20).

Various elements also emerged in the open-ended questions of the satisfaction questionnaire (see Figure 1 for the global results). The most frequently reported aspect of the workshop program appreciated by PBTS (72%) and their parents (31%) was the opportunity to hear the testimonial from a PBTS or a parent who had transferred to the adult care setting. In the PBTS group, a participant explained, “I really loved everything about the workshop. However, the presence of a testimonial was a very important and interesting part because it really clarified my vision of the adult hospital and its functioning”.

The aspects of the workshops that were least appreciated by PBTS were equally the presence of long and basic topics (27%) and the sound quality of the videoconference workshops (27%), while for parents, it was dissatisfaction with the virtual format (35%). In this regard, a parent said, “It is great to be able to access these workshops via zoom when we are from a remote area. However, it is more difficult to have contact with the other participants”.

### 3.3. Preliminary Efficacy

#### Results of the Transition Readiness Assessment Questionnaire (TRAQ)

The results regarding the impact of the targeted workshops for PBTS and for parents are detailed in Table 4. Effect sizes were generally in the expected direction but small. Although not statistically significant, an improvement in PBTS TRAQ scores was observed from the point of view of PBTS (t (9) = −1.14, *p* = 0.14, d = 0.36) as well as their parents (t (8) = −0.75, *p* = 0.24, d = 0.25). A statistically significant improvement following the workshops was only observed for PBTS self-efficacy (t (9) = −2.079, *p* = 0.03, d = 0.66), with a moderate effect size.

## 4. Discussion

Transition readiness needs are significant and diverse among PBTS and their families [5,12,31]. The quality of their transition readiness has a significant impact on their long-term health status and quality of life [18,21]. Therefore, there is a need to create and evaluate transition interventions addressing their specific needs [22,24,31,43,44]. The present study aimed to address this gap by co-developing and evaluating the feasibility and acceptability of targeted transition workshops among PBTS and their parents, as well as exploring the preliminary impact of the intervention on relevant transition readiness outcomes.

Regarding feasibility, the results indicated an achievement of pre-established retention objectives. Fifty percent of dyads participated in all three workshops (target 50%), and 83% of dyads participated in at least two workshops (target 60%). Our recruitment rate (38%) was similar to those observed in other intervention studies in pediatric cancer [42,60]. It was interesting to observe that 36% of families expressed interest without ultimately consenting to participate. This finding could potentially indicate difficulties in dyad recruitment (one or both of the parent–child dyads ultimately not wanting to participate). Regarding reasons for active refusal, some families mentioned that preparation for healthcare transition was not perceived as important or urgent, particularly by PBTS. It appears important that greater awareness of the importance of transition readiness be integrated into PBTS standard care [19,62]. Finally, some families declined to participate due to lack of time or scheduling difficulties. Future versions of the intervention could be offered in webinar format to address this barrier. Notably, no families were recruited through in-clinic or virtual recruitment strategies with partner community organizations. More efforts are needed to understand how to best engage PBTS and their caregivers in intervention research. In this regard, the research team observed that during the telephone and in-person recruitment at the hospital, the possibility of explaining the concept of healthcare transition readiness was helpful in engaging participants.

Regarding the objective of studying the acceptability of the intervention, the overall satisfaction target set at 75% of participants was reached for parents (86%) and was slightly below the target for PBTS (73%). There was notably a very low dissatisfaction response, particularly among PBTS, who often indicated a “neutral” satisfaction. The low dissatisfaction rate suggests that the intervention offers a well-built foundation for further development. The high satisfaction for the second workshop regarding social skills for both PBTS and parent groups concords with the literature indicating that parents perceived the sequelae on social skills and peer relationships to be the most devastating for youth and aligns with the expressed need by PBTS for psychosocial components in interventions intended for transition readiness and survivorship [40,63,64,65]. The overall average satisfaction of PBTS was most impacted by the final workshop on cognitive functioning, by the satisfaction level, which appeared lower (61%) than the other two workshops (77%; 79%). This workshop should, therefore, be particularly revised for young people. One avenue for reflection would be to integrate greater implications of PBTS and parents post-transfer, as their testimonials were the most appreciated element, particularly among the PBTS. In addition, it would be possible to reduce the time devoted to psychoeducation, which would also address the comments of PBTS, who appeared to have appreciated any lengthy periods presenting basic information less.

Regarding exploring preliminary effect indicators of the intervention, statistical analyses were ultimately limited by the small sample size. Nevertheless, there were some indications based on the direction of effect sizes, which indicated improvement in the expected direction. Based on the PBTS self-report and parent proxy report, PBTS appeared to have improved on average by approximately five points on the TRAQ measure post-intervention. Indeed, the increase of one point on the TRAQ scale is promising and shows a significant improvement in transition readiness, whether it is simply having developed an interest in learning the skill (2 on the Likert scale from 1 to 5) or having started to learn the skill (3 on the Likert scale ranging from 1 to 5), which are important steps that precede mastery of a skill (5 on the Likert scale from 1 to 5). These results are also consistent with the Obesity-Related Behavioral Intervention Trials (ORBIT) model, which describes the observation of a clinically significant difference as a key milestone to achieve for a preliminary study of an intervention [47]. A significant increase was found in PBTS’ sense of self-efficacy, which suggests that the intervention promoted the development of confidence in their ability to handle new situations, an important element for transition readiness.

Several strengths of the study can be highlighted. First, this intervention was co-developed and co-administered by clinicians from different professions as well as patient and parent partners. Their involvement made it possible to create an intervention that addresses multiple facets of transition readiness and aligns with the real-world needs of PBTS and their families. Secondly, different components of the workshop were shown to be both a strength and a distinguishing factor compared to other interventions in the literature (i.e., testimonials, involvement of parents as well as the young patient) [18,21,23,40,41]. Notably, the experiential knowledge of patient partners was a significant element of satisfaction among participants, particularly among PBTS, and therefore, it is important to consider this in future transition interventions. Furthermore, parents were also a focus of the intervention, which is consistent with the literature that indicates the need for parents to be included and more informed in the transition process and is aligned with recommendations to include them as well as the young patient [12,32]. Thirdly, we aimed to make the intervention accessible, such as offering a hybrid format and the payment of parking fees to reduce the costs associated with participation in person. These aspects are also essential in the context of the rarity of pediatric brain tumors in the general population and may live far from tertiary treatment centers where interventions are often offered [1]. Finally, the creation of an intervention manual, as well as materials for participants and facilitators, facilitates the possibility of replication and future access to the intervention for research as well as clinical implementation.

Some limitations of this study are also to be discussed. The first limitation concerns the sample size and the limited generalization of the results that can result from it. The small sample size may bias the portrait drawn on the feasibility of the intervention and limit the interpretation of the results. Notably, difficulties were encountered in the process of recruiting families, as no families were recruited outside the hospital despite virtual recruitment efforts. Although recruitment difficulties may be frequently encountered with this population [66], improvements will be necessary to the recruitment methods in order to obtain larger sample sizes. This could be a further opportunity to partner with PBTS project partners and seek their feedback on how to best engage participants, even regarding this early stage of recruitment. Another limitation is that the intervention was limited to the pediatric setting, both from an intervention perspective and outcome measures. Attention to the post-transfer environment will be necessary to consider the impact of interventions aiming to better support transition readiness. Furthermore, without a control group, it is not possible to distinguish with certainty whether the results represent an effect of time or an effect of the intervention. We remain encouraged by the preliminary indicators of positive change, but further study with larger samples is needed for more definitive conclusions. Finally, while the hybrid format allows for improved accessibility, some negative comments about the online experience were raised, which was the modality used by most workshop attendees. This observation, therefore, suggests that it would be necessary to maintain virtual participation as an option and further develop the quality of this experience. One way would be to increase the possibilities of interaction of participants in different forms and ensure the availability of heightened videoconference equipment.

## 5. Conclusions

In conclusion, this study describes the results of a novel pilot intervention tailored to the specific transition needs of PBTS. The results offer preliminary indicators of success, as well as opportunities for further refinement and development. There is also a need to test a revised intervention in larger samples and measure outcomes over longer periods. Given the importance of transition readiness as well as the risk of social and cognitive challenges present in other pediatric chronic illness groups (e.g., sickle cell disease, epilepsy), there is also an opportunity to expand the application of the intervention [22,29,67,68]. Finally, we note that workshops alone will not be sufficient to address the transition readiness needs of PBTS and their caregivers. Rather, it can be a complement to a more integrated transition preparation program provided over many years by the healthcare setting, such as ongoing assessment and support to develop autonomy skills, involvement and discussions with health professionals, follow-up of young people in the adult environment, or awareness of the adult environment [29].

## Figures and Tables

**Table 1:** Content of the targeted workshops for PBTS and their parents.

	1st Workshop	2nd Workshop	3rd Workshop
**Theme**	Self-management of the disease	Social Skills and Peer Relations	Cognitive challenges and return to daily activities
**Facilitators’ profession**	Nurse and clinical psychologist	Social worker and clinical psychologist	Occupational therapist and clinical psychologist
**Psychoeducation**	Differences between pediatric and adult settings	Importance of social relationships in adolescence, social skills in medical and social context	Overview of academic and career resources (education plans, scholarships, funds, work placement programs) and how to access them
**Targeted TRAQ Skills**	- Medication management (filling a prescription, reading medication labels). - How to share a personal health history	- How to ask questions, what questions to ask - How to assert your needs [45]	- How to learn and access services to manage daily activities (how to request resources adapted to school and work) - How to plan and organize daily activities

**Table 2:** PBTS socio-demographic and medical data.

	PBTS (n = 12)
	n (%)
Gender	
Male	9 (75)
Female	2 (17)
Neutral/non-binary	1 (8)
Type of tumor	
Astrocytoma	5 (42)
Craniopharyngioma	2 (17)
DNET	1 (8)
Ependymoma	4 (33)
Type of treatment	
Radiotherapy	6 (50)
Chemotherapy	1 (8)
Surgery	11 (92)
Endocrinology	1 (8)

**Table 3:** Mean percentages of participants’ responses to the satisfaction questionnaire (N = 20).

	PBTS		Parents	
	Acceptable and Very Acceptable (%)	Neutral, Acceptable, and Very Acceptable (%)	Acceptable and Very Acceptable (%)	Neutral, Acceptable, and Very Acceptable (%)
Workshop 1	77 *	98	83 *	98
Workshop 2	79 *	96	88 *	99
Workshop 3	61	98	88 *	96
Total average	73	97	86 *	98

* Achievement of the target set at 75%.

**Table 4:** Comparisons of pre-workshop and post-workshop results with *t*-test on dependent samples (N = 19).

	PBTS (n = 10)			Parents (n = 9)		
	Pre M (SD)	Post M (SD)	d	Pre M (SD)	Post M (SD)	d
TRAQ	59.80 (12.03)	64.40 (11.84)	0.36	64.56 (12.30)	69.22 (17.04)	0.25
Assertiveness	19.50 (4.30)	20.50 (4.45)	0.28	26.33 (4.30)	26.33 (3.91)	0.00
Planning	22.20 (5.76)	22.60 (5.30)	0.08	21.67 (7.02)	21.44 (7.42)	0.06
Self-efficacy	32.40 (5.68)	34.70 (4.67)	0.66 *	--	--	--
Parental overprotection	--	--	--	13.56 (1.24)	14.11 (1.36)	0.15

* *p* < 0.05.

## Data Availability

Data presented in this article are not readily available because participants did not consent for their information to be publicly shared.

## Supplementary Materials

The following supporting information can be downloaded at: https://www.mdpi.com/article/10.3390/curroncol32010034/s1, Workshop satisfaction questionnaire.
