# Supplementary material for: Targeted Transition Readiness Workshops for Pediatric Brain Tumor Survivors: Feasibility, Acceptability, and Preliminary Effects
Source: Curr Oncol. 2025 Jan 8;32(1):34. doi: 10.3390/curroncol32010034 (PMC11763518; doi:10.3390/curroncol32010034)
Supplement: Supplementary file 1 [file curroncol-32-00034-s001.zip › curroncol-3398814-supplementary.pdf]

Participant Code: __________

Workshop satisfaction survey (English)

The topic of the workshop was:
- ☐ Care management
- ☐ Social relations
- ☐ Cognitive/academic functioning

I participated:
- ☐ Face-to-face
- ☐ Visuoconference (Teams or Zoom)

	Strongly disagree	Disagreement	Neutral	All right	Strongly agree
The format of the workshop (face-to-face/virtual) was ideal					
The topic of the workshop was relevant					
The workshop was well structured					
I learned something new					
The information was useful					
The information was easy to understand					
The activities practiced were useful					
The speaker was well informed					
The discussion with the youth/parent post transfer was helpful					
The duration of the workshop was ideal					
The resources given are helpful					
I would recommend this workshop					

What I liked most about the workshop:

What I liked least about the workshop:

Other comments:

Évaluation de la satisfaction de l'atelier

Le sujet de l'atelier portait sur :

- ☐ La gestion des soins
- ☐ Les relations sociales
- ☐ Le fonctionnement cognitif/académique

J'ai participé :

- ☐ En présentiel
- ☐ En visuoconférence (Teams ou Zoom)

	Fortement désaccord	Désaccord	Neutre	D'accord	Fortement d'accord
Le format de l'atelier (présentiel/virtuel) était idéal					
Le sujet de l'atelier était pertinent					
L'atelier était bien structuré					
J'ai appris quelque chose de nouveau					
Les informations étaient utiles					
Les informations étaient faciles à comprendre					
Les activités pratiquées étaient utiles					
L'intervenant était bien informé					
La discussion avec le jeune/parent post transfer était aidant					
La durée de l'atelier était idéale					
Les ressources données sont aidantes					
Je recommanderais cet atelier					

Ce que j'ai le plus aimé à propos de l'atelier :

Ce que j'ai le moins aimé à propos de l'atelier :

Autres commentaires :
